# Enhancing Effects of Olaparib by Alpha- and Beta-Emitting Radionuclides, X-Rays, and Ultraviolet A Light in Combination with Ortho-IodoHoechst in a Prostate Cancer Cell Model

**DOI:** 10.3390/ph17111450

**Published:** 2024-10-30

**Authors:** Andrea C. Luna Mass, Roswitha Runge, Kerstin Wetzig, Lisa Huebinger, Claudia Brogsitter, Joerg Kotzerke

**Affiliations:** Department of Nuclear Medicine, University Hospital Carl Gustav Carus, Technische Universität Dresden, Fetscherstrasse 74, D-01307 Dresden, Germany; roswitha.runge@uniklinikum-dresden.de (R.R.); kerstin.wetzig@ukdd.de (K.W.); lisa.huebinger@uniklinikum-dresden.de (L.H.); claudia.brogsitter@uniklinikum-dresden.de (C.B.); joerg.kotzerke@uniklinikum-dresden.de (J.K.)

**Keywords:** prostate cancer, PC-3 cells, Olaparib, radionuclides, X-ray, ortho-iodoHoechst, UVA light, clonogenic survival, persistent DNA double-strand breaks

## Abstract

**Background:** New therapeutic strategies for metastatic castration-resistant prostate cancer (mCRPC) have been developed in the past to achieve the best response rates. Most recently, the use of combination therapies has been explored to optimize patient outcomes. Poly(ADP-ribose) polymerase inhibitors (PARPi) may help to treat mCRPC more effectively. **Objectives:** This study aimed to determine whether the combination of a PARPi with different radiation qualities results in different levels of radiosensitization of PC-3 cells. **Methods:** The radiosensitizing potential of Olaparib in combination with ^177^Lu, ^223^Ra, X-rays and photodynamic therapy (PDT) using the UVA light-activated photosensitizer ortho-iodoHoechst33258 (oIH) was evaluated by determining the clonogenic survival, DNA damage and cell cycle analysis. **Results:** Here, we show that this combination strategy differentially sensitized PC-3 cells to different radiation qualities. The combination of ^177^Lu with Olaparib increased the numbers of persistent double-strand breaks (DSBs) by a factor of 3.3 and cell death in PC-3 cells. Overall, the β-emitter ^177^Lu indicated a higher radiosensitization efficacy compared to ^223^Ra, with X-rays corresponding to dose modification factors (DMF) of 1.77, 1.17 and 1.16 respectively. Even in the case of the α-emitter ^223^Ra, the effects were much less pronounced than for ^177^Lu. PARPi also showed a slight potentiation of the cytotoxic effects both in co-treatment with X-rays and with PDT. **Conclusions:** The results of our study indicate a potential role for Olaparib in further optimizing the PSMA radioligand therapy (PRLT) outcomes. However, further evaluation of the combination of PARPi with PRLT is needed to gain more insights into improving the benefit to patients suffering from mCRPC.

## 1. Introduction

Prostate cancer (PCa) is the second most commonly diagnosed cancer worldwide, and metastatic castration-resistant prostate cancer (mCRPC) accounts for nearly all prostate cancer deaths in the USA [1]. PSMA-targeted ligands have been established in the diagnostics [2,3] and therapy of mCRPC. In particular, therapeutic delivery of the ^177^Lu-labeled PSMA-targeted ligands ^177^Lu-PSMA-617 [4] and ^177^Lu–PSMA-I&T [5] has been developed in the past. Clinical trials such as the VISION trial have demonstrated the high efficacy of targeted radiopharmaceutical therapies (TRT), such as ^177^Lu-PSMA-617 radioligand therapy (PRLT) for mCRPC [6], and the ALSYMPCA trial for the radionuclide targeted α-therapy (TAT) with ^223^Radium-dichloride (^223^Ra) selectively targeting bone metastases from prostate cancer [7]. Recently, α-therapy with ^225^Ac-PSMA-617 was added to the therapeutic spectrum for mCRPC [8].

Although a lot of the mCRPC patients may benefit from these therapies, a few of them have shown minimal or no response to PRLT. Therefore, there is a need to increase the efficacy of PRLT [9]. Combinatorial approaches are a potential strategy to improve patients’ outcomes without increasing the radiotoxic effects. Increased DNA damage has been observed in tumor cells following the combination of ionizing radiation with epigenetically active compounds [10,11]. The DNA repair-associated enzyme poly(ADP-ribose) polymerase-1 (PARP-1) is known to be required for the repair of single-strand breaks (SSB); PARP-1 also has a function in the repair of double-strand breaks (DSBs) and replication fork damage [12,13]. PARP-1-deficient (or inhibited) cells showed reduced rates of base excision repair (BER) [14] and hypersensitivity to agents that induce the formation of SSBs [15]. The molecular function of PARPi is based on the concept of synthetic lethality, where only the combination of PARP inhibition and the *Breast Cancer genes 1 and 2* (*BRCA1/2)* deficiency is lethal [12]. Further insights into the mechanistic basics of cytotoxic effects induced by various PARP inhibitors and new genetic repair pathways in tumor cells were provided by Murai et al. [16].

Several clinical studies have been designed to determine the therapeutic efficacy of PARPi in the treatment of *BRCA1/2* mutated cancers such as breast, ovarian, pancreatic and prostate, as summarized by Ragupathi et al. [17]. Importantly, for mCRPC patients with *BRCA1/2* or *ATM* mutations, the PROfound trial demonstrated a longer progression-free survival compared to either enzalutamide or abiraterone [18]. The combination of ^177^Lu-PSMA-617 and Olaparib is currently enrolling patients in the phase 1 LuPARP trial regardless of their *BRCA* mutation status (NCT03874884).

Currently, the combination of ^177^Lu-targeted radiotherapy with a PARPi, most commonly Olaparib, has shown a successful enhancement of cancer cell killing in neuroendocrine tumors (NETs) [19,20,21]. Although numerous preclinical and in vitro studies have focused on the radiosensitization of tumor cells by Olaparib in combination with β-emitters [22] or X-rays [23,24], systematic studies on Olaparib-mediated radiosensitization by combination with TRT are required. To date, there are only few studies on radiopharmaceuticals compared to external radiation sources.

In this study, ^177^Lu- or ^223^Ra radioactive solutions were used for irradiation of the PC-3 cells. The β-emitter ^177^Lu is characterized by low linear energy transfer (LET) and mainly causes radical-mediated DNA damage. In addition, the α-emitter ^223^Ra (high LET) was utilized, which causes complex DNA double-strand breaks that are mostly repaired by the homologous recombination repair (HRR) pathway and partially by non-homologous end joining (NHEJ) [25,26]. Because of its frequent use in cell toxicity studies, external X-ray-irradiation was also used to evaluate the Olaparib-modulated increase in tumor cell killing. In addition, we tested a phototherapeutic approach based on the concept of photodynamic therapy (PDT) [27] using the photosensitizer ortho-iodoHoechst (oIH) activated by UVA light.

Assuming possible differences in the response to these combination therapy approaches regarding different types of radiation qualities and to assess the radiosensitizing potency of the PARPi Olaparib, we conducted this in vitro study in prostate cancer cells.

The aim of this study was to evaluate whether the PARPi Olaparib in combination with ^177^Lu, ^223^Ra, X-rays, and UVA light in the presence of oIH is able to enhance the cell killing effects in PC-3 cells. To evaluate the cytotoxic effects of these combinatorial approaches, we used common biological endpoints, such as clonogenic cell survival, residual DSB by the γH2A.X foci assay, and cell cycle distribution.

## 2. Results

### 2.1. Cytotoxicity of Olaparib Determined by Clonogenic Survival of PC-3 Cells

**Figure 1:**
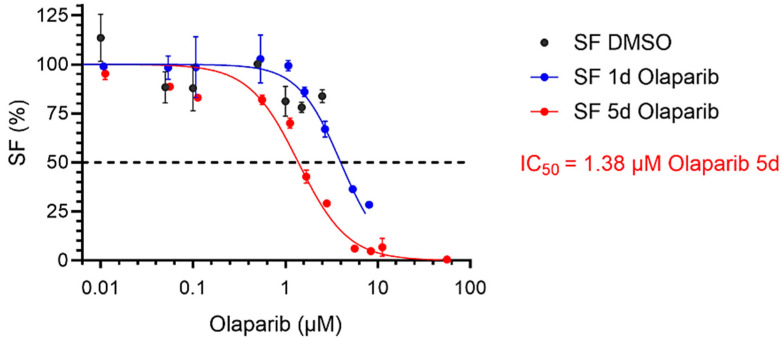
Cytotoxicity of Olaparib in PC-3 cells in the concentration range from 0.01 to 50 µM incubated for 1 day or 5 days. DMSO equivalent concentrations (without Olaparib) are included for reference. Data show the mean ± SD. Curves were fitted with non-linear regression by GraphPad 10.2.3.403.

To determine the PARPi concentration for the combination experiments, the PC-3 cells were treated with Olaparib at concentrations ranging from 0.01 to 50 µM for one day or five days incubation periods. The chemotoxicity of the drug was evaluated using the clonogenic survival assay. At a concentration of 1 µM Olaparib and a treatment period of 5 days, the survival fraction was reduced to a SF value of 70.03 ± 2.5% compared to untreated control cells. This indicates moderate chemotoxicity at this concentration. The IC_50_, i.e., the inhibitory concentration required to reduce cell survival by 50%, was 1.38 µM after 5 days of incubation with Olaparib; the corresponding IC_50_ value for the incubation period of 24 h was 3.97 µM (Figure 1).

### 2.2. Dose Response Curves of Combined Treatments with Olaparib and ^177^Lu, ^223^Ra, X-Ray and oIH Activated by UVA

To investigate wether PARPi sensitizes the PC-3 cells to ^177^Lu, ^223^Ra, X-ray or ortho-iodoHoechst plus UVA exposure, we determined the cell survival. To elucidate the different effects of the radiation qualities sensitized by Olaparib, we calculated the respective doses required to reduce the surviving fractions (SFs) to 37% (D_37_-values) (see Table 1).

**Figure 2:**
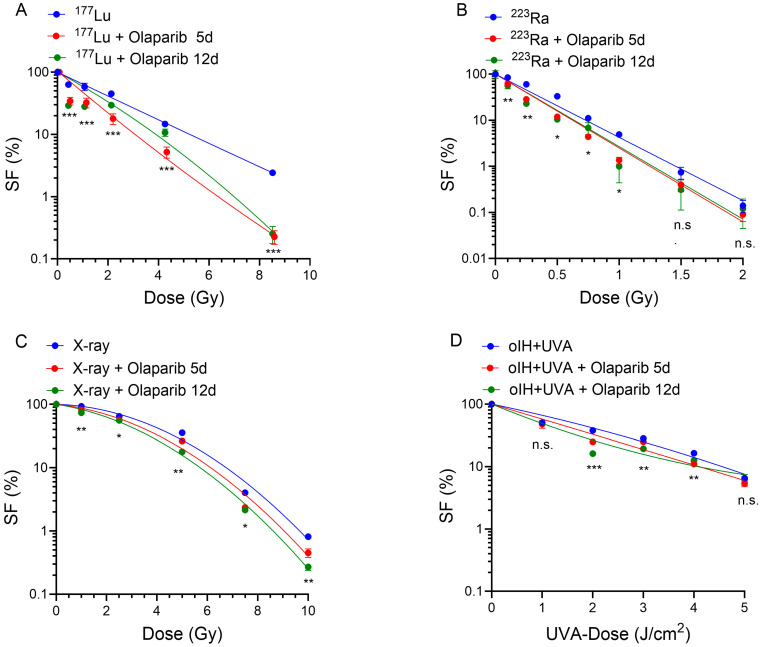
Olaparib treatment for 5- or 12-days sensitized PC-3 cells to different radiation qualities. Survival curves of single and combined treatment experiments with Olaparib and (**A**) ^177^Lu (**B**) ^223^Ra, (**C**) X-rays and (**D**) oIH + UVA are shown. Results are expressed as mean SF ± SD. Statistically significant differences are plotted in the graph, *** *p* < 0.0001, ** *p* < 0.001, * *p* < 0.01. Asterisks and brackets indicate significant differences for 5 days of Olaparib treatment in response to irradiation alone at *p* < 0.05, n.s. = not significant. Curves are fitted by the Linear Quadratic Model (LQM), linear (**B**) or linear quadratic (**A**,**C**,**D**) curve fits, GraphPad 10.2.3.403.

Significant differences in the SFs were observed when comparing the ^177^Lu monotherapy and the Olaparib-modulated samples over 5 and 12 days at each of the dose points (Figure 2A). When comparing the Olaparib treatment times, significant differences were found only at the 0.4 and 1.0 Gy ^177^Lu dose points (*p* ≤ 0.05).

The effects of concurrent PARPi treatment of the PC-3 cells irradiated with α-radiation by ^223^Ra showed a significant reduction in the SF compared to ^223^Ra monotherapy (SF_223Ra_ = 84.01% vs. SF_223Ra+Olap5d_ = 62.14%, *p* = 0.031 at 0.1 Gy and SF_223Ra_ = 60.16% vs. SF_223Ra+Olap5d_ = 28.46% at 0.25 Gy, *p* = 0.011). With increasing ^223^Ra doses a decreasing cell killing effect was observed. Overall, the sensitizing effect of Olaparib was less pronounced with ^223^Ra compared to the β-emitter ^177^Lu. Longer treatment periods with Olaparib (5 days vs. 12 days) showed significant differences in the cells irradiated with 0.25 Gy ^223^Ra. At higher dose points, there was no correlation between longer incubation of the PARP inhibitor and a change in the surviving fractions.

Looking at the dose responses for X-rays, the combined treatment with Olaparib for 5 days resulted in a statistically significant sensitization of PC-3 cells to radiation at each of the dose points (Figure 2C). For example, when comparing 5 Gy X-ray radiation alone with 5 Gy X-rays co-treated with Olaparib, the corresponding SF-values were reduced from 35.53% to 26.3% (*p* = 0.0054). An extension of the Olaparib incubation period from 5 to 12 days led to a significant decrease in the SF only at 5 Gy and 10 Gy.

Figure 2D shows the results of PC-3 cells treated with UVA light in the range of 1–5 J/cm^2^ and oIH at a constant concentration of 0.0075 µM without and with Olaparib modulation. The combined treatment of PC-3 cells (Olaparib for 5 days) showed statistically significant differences at irradiation doses of 2.0–4.0 J/cm^2^. Extending the incubation period from 5 to 12 days resulted in significant differences between the respective SFs at UV doses of 2.0 J/cm^2^ and 3.0 J/cm^2^, suggesting that the PARPi caused replication fork arrest and lethal DSB that were more pronounced after 12 days Olaparib treatments.

Furthermore, to quantify the sensitizing contribution of Olaparib after 5 days of incubation, dose-modifying factors (DMF) were calculated as the ratio of the D_37_-values from the regression analysis for irradiation in the absence (D_37_(–Olaparib)) and presence (D_37_(+Olaparib)) of Olaparib for each radiation quality. Considering the sensitizing effects induced by Olaparib co-treatment over 5 days, the highest efficacy was found for the combination with ^177^Lu (DMF = 1.77), (see Table 1).

Overall, among the radiation qualities the β-radiation of ^177^Lu lead to the highest radiosensitizing effects in co-treatment with Olaparib over 5 days when comparing the DMF at D_37_. However, these effects were dose dependent. In particular, the extension of Olaparib treatment onto 12 days supports the assumption of PARPi caused replication fork arrest and lethal DSB that were more pronounced after prolonged Olaparib treatment.

### 2.3. Persistance of DSB After Combined Treatments with Olaparib

**Figure 3:**
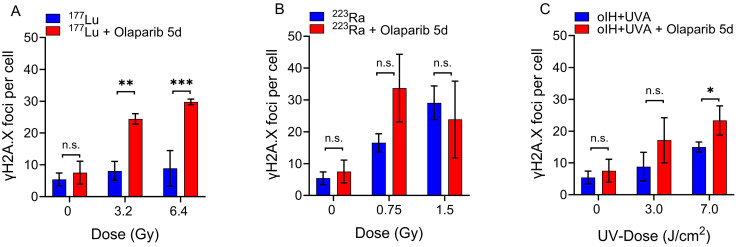
Co-treatment with Olaparib for 5 days leads to radiosensitization of PC-3 cells. The numbers of γH2A.X foci per cell after irradiation alone or after combined treatments with Olaparib (1 µM) over 5 days for (**A**) ^177^Lu, (**B**) ^223^Ra and (**C**) oIH + UVA are displayed. Asterisks and brackets indicate significant differences at *p* < 0.05 * *p* < 0.05, ** *p* = 0.001, *** *p* < 0.0001, n.s. = not significant. The mean values ± SD of 3 individual experiments are shown.

To evaluate the effect of Olaparib on the DSB processing, the PC-3 cells were irradiated with ^177^Lu, ^223^Ra or UVA light in combination with oIH to determine whether or not, the addition of 1 µM Olaparib has a radiosensitizing effect on the cells (Figure 3).

First, we found that Olaparib did not cause a significant increase in DSB induction in the non-irradiated PC-3 cells (5.41 foci/cell in the absence of Olaparib vs. 7.52 foci/cell in the presence of 1 µM Olaparib, *p* = 0.425).

Second, a comparison of the DNA damaging effect of the combined treatment with ^177^Lu and Olaparib revealed a higher radiosensitizing potential of the combination than ^177^Lu alone by a factor of 3.3.

Third, as expected, a high number of DSBs was observed at high doses of α-irradiation as a single agent. Treatment with Olaparib did not induce an increase in γH2A.X foci at 1.5 Gy ^223^Ra (29.11 foci/cell without Olaparib vs. 23.87 foci/cell with Olaparib, *p* = 0.26). Note that the effect of Olaparib was dose dependent, as Olaparib increased the number of γH2A.X foci at 0.75 Gy in contrast to 1.5 Gy where the combined treatment showed lower levels of DNA damage than ^223^Ra alone (Figure 3B).

Finally, when UVA irradiation was combined with oIH, a significant increase in the number of γH2A.X foci was observed only at the dose of 7.0 J/cm^2^ (14.97 foci/cell without Olaparib vs. 23.39 foci/cell with Olaparib, *p* = 0.039). A nearly three-fold increase in DSBs was also observed at the 3.0 J/cm^2^ dose. However, the results at 3.0 J/cm^2^ were not statistically significant (*p* = 0.162).

Altogether, ^177^Lu co-treated with Olaparib over 5 days led to the highest radiosensitizing efficacy in this assay compared to ^223^Ra and oIH + UVA light.

The combined approach using alpha radiation of ^223^Ra led to a two-fold increase of persisting γH2AX-foci only at 0.75 Gy, whereas oIH + UVA light showed a nearly three-fold increase of persisting γH2AX-foci (Table 2).

**Figure 4:**
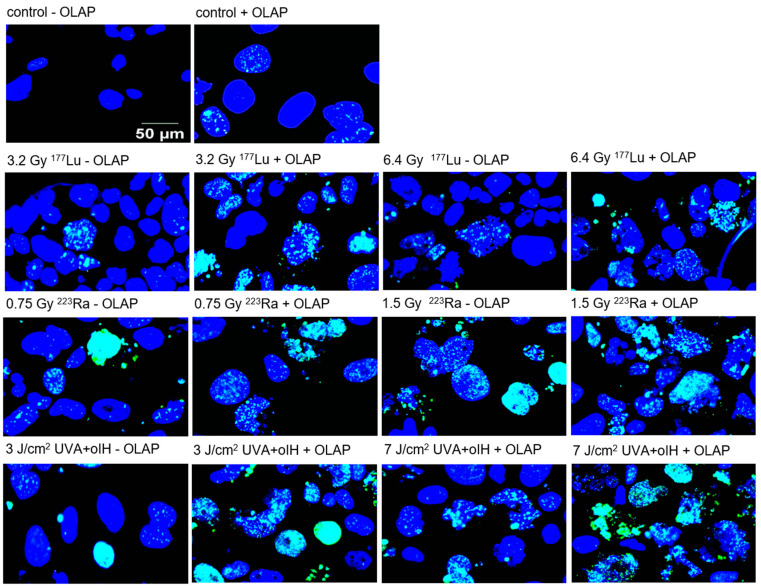
Representative images of PC-3 cells after mono- or combination therapy with ^177^Lu, ^223^Ra or oIH plus UVA ± Olaparib (1 µM) incubated for 5 days. Untreated and Olaparib-treated samples are included as controls. Immunofluorescent staining against γH2A.X foci (green) and nuclear staining (blue) as merged images are shown (bar = 50 µm).

Figure 4 presents images of γH2AX foci patterns in PC-3 cells after exposure to ^177^Lu, ^223^Ra and oIH plus UVA and Olaparib for 5 days. In particular, the ^223^Ra treated PC-3 cells displayed nuclei with closely spaced foci, suggesting clustered DNA damage.

### 2.4. Radiosensitizing Effects of Olaparib on Cell Cycle Distribution and Sub-G1 Fraction

The aim of these experiments was to determine whether Olaparib co-treated with different radionuclides has an enhancing effect on the accumulation of cells in the S and G2/M checkpoints of the cell cycle.

To investigate the effects of α- and β-irradiation, PC-3 cells were co-treated with Olaparib (1 µM) with ^177^Lu at doses of 3.2 or 6.4 Gy or with ^223^Ra at 0.75 and 1.5 Gy for 24 h, followed by another 5 days of incubation with Olaparib.

Administration of 1 µM Olaparib as a single agent resulted in only a negligible (non-significant) increase in the number of cells in S and G2/M phase compared to the control sample.

**Figure 5:**
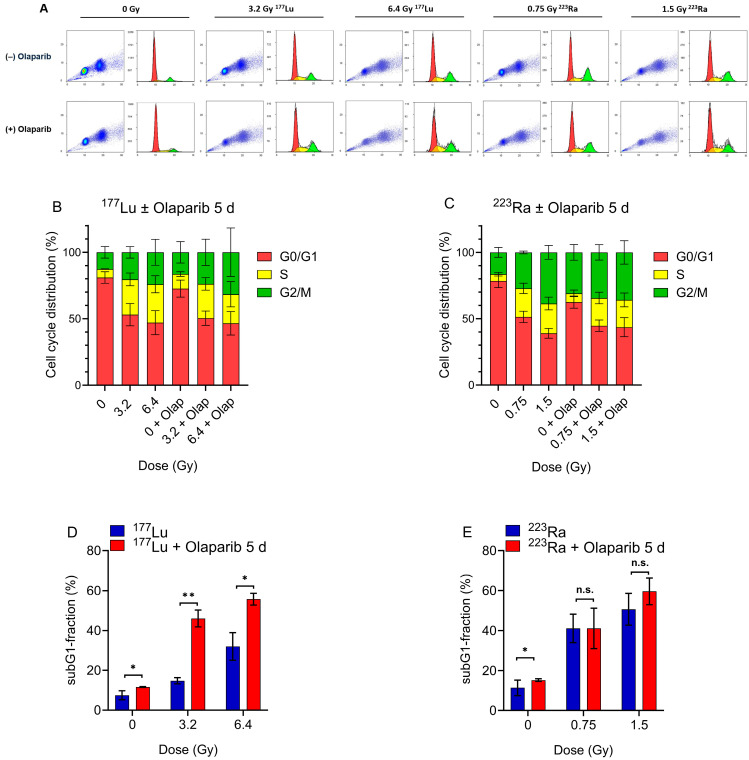
(**A**) Cell cycle distribution of PC-3 cells after irradiation with ^177^Lu- or ^223^Ra ± 1 µM Olaparib (1 event = 1 cell). Histograms and dotblots of ^177^Lu or ^223^Ra exposed PC-3 cells in the absence (upper panel) or presence of Olaparib (lower panel) are displayed (FlowLogic™ version 8.7). The x-axes show the PI fluorescence signals, the y-axes show the counts contributing to cells. Red, yellow or green color indicate the G0/G1, S or G2/M phase histogram peaks, respectively. (**B**,**C**) Distribution of cell cycle phases after irradiation with ^177^Lu- or ^223^Ra ± 1 µM Olaparib for 5 days. The mean values ± SEM (n = 3) are shown. For clarity, the significant differences are not marked in the graph. (**D**,**E**) Column graphs of Sub-G1 fractions display the mean values ± SEM (n = 2), * *p* < 0.05, ** *p* < 0.005, n.s. = not significant.

Results after ^177^Lu monotherapy showed an increase in the percentage of cells in the S phase and, to a lesser extent, in G2/M. After irradiation with 3.2 Gy or 6.4 Gy ^177^Lu single treatment, an approximately four-fold higher number of cells in the S phase was observed compared to the control at 3.2 Gy (*p* = 0.0004) and at 6.4 Gy (*p* = 0.029); see Figure 5B.

With the dual treatment of ^177^Lu and Olaparib, only slight differences in the S and G2/M phases were analyzed compared to the ^177^Lu monotherapy. These differences were not statistically significant.

In contrast to ^177^Lu, a significant increase in the G2/M phase after irradiation with ^223^Ra alone lead to 27.22% and 38.63% G2/M percentages at 0.75 Gy and 1.5 Gy, respectively, compared to 16.53% observed in non-irradiated cells. Furthermore, Olaparib in combination with ^223^Ra irradiation induced a slight cell activation into the S or G2/M checkpoint in PC-3 cells. The dose escalation of ^223^Ra did not result in increased arrest in S or G2/M.

Overall, ^177^Lu monotherapy induced the migration of cells in the S phase, whereas ^223^Ra induced mainly G2/M arrest. Additional treatment with Olaparib only slightly increased these effects.

It should be noted that only cells that survived or had sufficient genetic integrity were included in the cell cycle analysis. Our analysis of the Sub-G1 phase showed a decreasing number of cells entering the cell cycle at higher radiation doses and in combination with Olaparib. Therefore, in these experiments, the Sub-G1 population was examined to determine whether 1 µM Olaparib increased the proportion of cells with reduced DNA content compared to monotherapy with ^177^Lu or ^223^Ra. Comparing the combination of 3.2 Gy ^177^Lu + 1 µM Olaparib to irradiation with 6.4 Gy ^177^Lu without Olaparib, the results indicate that the combination was as effective as radiation with 6.4 Gy ^177^Lu alone (Figure 5D). This observation suggests that the PARP inhibitor Olaparib is able to enhance the effect of the β-emitter, such that a significant reduction of up to 50% of irradiation dose is required to achieve comparable results.

A significant difference in the in the Sub-G1 fractions between irradiated cells and those co-treated with Olaparib was found at 3.2 Gy of ^177^Lu, by a factor of 3.13 (14.79% without Olaparib vs. 46.01% with Olaparib, *p* = 0.0034). At 6.4 Gy, the dual treatment showed a ≈1.8-fold increase in efficacy (31.98% without Olaparib vs. 55.7% with Olaparib, (*p* = 0.01).

In experiments with ^223^Ra alone, a significant increase in the Sub-G1 population was observed at 0.75 Gy, indicating an increase by a factor of 2.6 compared to control cells. Similarly, a more than four-fold increase was observed at 1.5 Gy. Looking at the dual-treated samples, at 0.75 Gy ^223^Ra, the number of cells in the Sub-G1 phase increased from 29.73% without Olaparib to 41.1% with Olaparib (*p* = 0.050). At higher doses, the radiosensitizing effect of Olaparib was less pronounced (*p* = 0.059). Overall, the results of the Sub-G1 analysis show a greater effect of Olaparib in combination with ^177^Lu compared to ^223^Ra (Table 3).

In summary, the cell cycle analysis achieved comparable effects for ^177^Lu and ^223^Ra co-treated with Olaparib; however, at higher doses, these effects became stronger again for ^177^Lu (Table 3). Similarly, the sub-G1 analysis indicated a higher efficacy for ^177^Lu compared to ^223^Ra.

## 3. Discussion

The aim of this study was to investigate whether the PARP inhibitor Olaparib in combination with different radiation sources induces sensitizing effects in *BRCA1/2*- wild-type PC-3 cells. Several in vitro studies have shown encouraging results in various tumor cell models by combining external irradiation with PARP inhibitors [23,28,29]. However, there are only a few studies on PARP inhibitors that have sensitized tumor cells to targeted radionuclide therapies, and PSMA targeting studies have rarely been published [22,30,31,32].

In this study, we demonstrated that the PARPi Olaparib could sensitize PC-3 cells when simultaneously treated with different radiation qualities. Consistent with our hypothesis, the clonogenic survival assays indicated that the efficacy of the combinatorial approaches depends on the type of irradiation. Specifically, the radiosensitizing effect was most pronounced when the β-radiation of ^177^Lu was used in combination with Olaparib, compared to the other radiation sources.

The clonogenic survival results may indicate that the role of PARP-1 in SSB repair is particularly important for the low-LET emitter ^177^Lu compared to the high-LET emitter ^223^Ra. Since low-LET emitters induced more SSBs than DSBs (ratio 25:1), PARP inhibition impaired SSB repair, which, in part, led to the stimulation of DSB formation, that, in turn, increased the sensitivity to ionizing radiation. Their cytotoxicity to HRR-deficient cells could have been caused by replication fork collapse, which requires HRR [33].

Surprisingly, our results for low LET X-rays do not support this hypothesis. However, the main difference in the experimental setting was the irradiation time periods of the PC-3 cells between ^177^Lu (24 h) and X-rays (several minutes) to achieve the same energy doses. Since we are not sure of the reason for our findings, we identified the different dose rates as a possible explanation.

On the other hand, ^223^Ra induces primarily more complex DNA damage in the form of clustered DSBs, which are highly destabilizing for chromatin and compromising for their successful repair [25], because repair pathways such as NHEJ are suppressed. Overall, the interaction of PARPi and NHEJ remains controversial [25,33].

In summary, we conclude that the enhanced cytotoxicity of the β-radiation of ^177^Lu in combination with the PARPi Olaparib is attributable to their higher proportion of SSB formation compared to a higher proportion of primarily occurring, complex DSB that are induced by ionizing radiation of high LET emitters such as ^223^Ra [25]. In that case, the mechanisms of the PARPi impairing the BER are of rather minor importance.

Our observations from clonogenic survival assays are at least partially consistent with the results reported by Hirai et al. [34]. We observed similar dose-modifying factors for external X-rays and the high-LET emitter ^223^Ra (1.2 for both), and Hirai et al. reported enhancement ratios of 1.4 for γ-rays and high-LET carbon ion beam radiation for both. In our study, the low-LET emitter ^177^Lu led to more pronounced sensitizing effects by Olaparib 1 µM (DMF = 1.8). In contrast to Hirai et al., we found only a dose-dependent increase in damage in the cell cycle distributions but no significant effect of Olaparib in this assay. Additionally, we demonstrated similar radiosensitizing effects of Olaparib for ^177^Lu compared to Tesson et al. for ^131^I-MIP-1095 in LNCaP spheroids [24]. In agreement with recent studies regarding Olaparib combined with X-rays [23,29], we have demonstrated here that Olaparib sensitized PC-3 cells to X-rays. Dose-dependent effects were observed when Olaparib was co-administered with X-ray or γ-radiation [29,35].

Furthermore, Bannik et al. found that the DNA repair inhibitors *ATM* and DNA-PK sensitized cells to ^223^Ra to a lesser extent than the combination with X-rays [36]. This is only partially consistent with our results since we found very similar dose-modifying factors for ^223^Ra and X-rays with the PARPi Olaparib.

The results of the photodynamic approach showed a significant cytotoxic effect of Olaparib compared to oIH plus UVA alone by a factor of 1.25, which is similar to the factors determined for ^223^Ra and X-rays. Upon irradiation with UVA light, the ortho-iodo-Hoechst molecule acts as a photosensitizer, as the production of highly reactive ligand radicals causes DNA damage, predominantly SSB [37].

To further evaluate the potential of the combination therapy, we quantified the number of γH2A.X foci after 5 days of Olaparib treatment as a measure of persistent DSBs. In contrast to Ruigrok et al. for ^177^Lu-PSMA in PC-3-PIP cells, we found a clear effect of Olaparib in combination with ^177^Lu. Compared to ^177^Lu monotherapy, the number of γH2AX foci per cell increased three-fold in the combinatorial setting of our experiments. These findings correspond to those of Rauch et al., who also found decreased clonogenic survival and higher amounts of persistent DNA damage (γH2AX foci) for combined treatment with ^177^Lu-DOTATOC and PARPi compared to the single treatment with Olaparib [20].

The combination with ^223^Ra resulted in a higher number of persistent γH2AX foci per cell compared to ^223^Ra monotherapy, but only at the low dose point (0.75 Gy); at 1.5 Gy, the Olaparib effect disappeared. As mentioned above, the repair of clustered DSBs induced by α-particles is more complex than DSB repair after exposure to γ- or β-radiation. Furthermore, the identification of individual foci can be complicated by the overlapping of foci in the nuclei [38].

In addition, the combination of oIH and UVA demonstrated an Olaparib-induced increase in DSBs and a decrease in cell survival. A study conducted by Tanaka et al. showed that the PDT with talaporfin and co-treatment with PARP-1 inhibitors, such as Olaparib, enhanced the efficacy of PDT in gastric cancer cells (MKN45) examined by DSB induction [25,39].

Looking at the results of cell cycle analysis, the ^177^Lu monotherapy induced a significant dose-dependent migration of PC-3 cells into the S phase, whereas a significant distribution into the G2/M phase was observed after irradiation with ^223^Ra alone. Although additional treatment of ^177^Lu or ^223^Ra with Olaparib slightly increased the effects, these differences did not reach statistical significance. These results suggest a rather minor role of Olaparib under our experimental conditions, including a 5-day incubation period after irradiation.

In contrast to our results, several studies reported a higher degree of cell cycle arrest in G2/M by PNKP or *ATM* inhibition for ^223^Ra particle therapy than for low-LET radiation [26,40]. Further, Nonnekens et al. demonstrated an increase of the number of neuroendocrine tumor cells in G2/M phase, treated with ^177^Lu-DOTA-TATE and Olaparib for 6 days [30]. In agreement with our results for ^223^Ra mono-treatment, the study by Wang et al. showed a significantly increased distribution of PC-3 cells in G2/M phase after carbon ion irradiation compared to X-rays [41].

Therefore, we decided to determine the Sub-G1 fractions of PC-3 cells, which are characterized by reduced DNA content indicating apoptotic processes [42]. These results showed significant cytotoxic effects of the Olaparib treatment compared to radiation alone for ^177^Lu. Both ^223^Ra monotherapy and ^223^Ra plus Olaparib induced higher Sub-G1 fractions of PC-3 cells than ^177^Lu. Overall, when looking at these results, it was evident that a significant number of cells disappeared from the active cell cycle in the Olaparib-treated cells. This could be attributed to the Olaparib-induced increased number of cells that were highly fragmented or apoptotic. Colony formation analysis indicated that a significant proportion of cells in the Sub-G1 phase may have been associated with a reduced capacity for replication and a high degree of non-dividing cells.

Altogether, our results obtained from clonogenic survival revealed radiosensitizing potential when combining the PARPi with radionuclides, X-rays or PDT. These findings confirm our hypotheses that cytotoxic effects of the combinations varied depending on the type of radiation. As expected, we observed the highest radiosensitizing effects of Olaparib for the β-radiation of ^177^Lu, whereas the α-radiation of ^223^Ra caused cytotoxic effects to a minor extent than ^177^Lu. This is in line with other studies, as discussed above. An exception are our results of cell cycle analysis that could not confirm the radiosensitizing potential of Olaparib in contrast to other studies. However, as it is somewhat surprising that similar effects were found for ^223^Ra compared to X-rays, there is a need for further studies.

It should be noted that some of these effects were rather less pronounced, which could be attributed to certain limitations of this study. Here, we used PC-3 cells as a tumor model; however, comparative studies would be valuable to verify if the results are reproducible with other prostate cancer cell lines, such as DU-145 or LNCaP.

Furthermore, we are aware that the results found here are important for basic knowledge on combining a PARPi with radiotherapy, but the findings can only partly extrapolate into clinical use. This is because fundamental factors such as immune response, oxygenation and tumor heterogeneity cannot be evaluated with our current in vitro methods.

## 4. Materials and Methods

### 4.1. Cell Culture

The human prostate cancer cell line PC-3 was obtained from the American Type Culture Collection (ATCC^®^, Manassas, VA, USA, CRL-1435™) The cells were isolated from a bone metastasis of a poorly differentiated grade IV human prostate adenocarcinoma. The PC-3 cells belong to the small cell neuroendocrine prostate carcinoma (SCNC) which is characterized by the lack of the expression of prostate-specific antigen (PSA) and androgen receptor (AR) expression, making it therapeutically androgen-independent. PC-3 cells show high levels of neuroendocrine tumor markers such as chromogranin A (CgA) and neuron-specific enolase (NSE) [43]. The adherent growing cells were cultured in Roswell Park Memorial Institute (RPMI) medium supplemented with 10% (*v/v*) fetal calf serum (FCS) and 1% (*v/v*) non-essential amino acids (NEA). The culture medium was changed every 2–3 days. The cells show adherent growth as a monolayer, with a doubling time of approximately 30 h. Via polymerase chain reaction (PCR) tests, the cell line is proven to be free of mycoplasmas.

No somatostatin receptor subtype 2 (SSTR2) expression was found by immunostaining [11]. Evaluation of a *BRCA1/2* mutation by DNA sequence analysis using next generation sequencing (NGS) technology at the Institute of Pathology, University Hospital Dresden, Germany, revealed no pathogenic mutation indicating a *BRCA1/2* wild-type sequence.

### 4.2. Olaparib

Olaparib (AZD2281) was purchased from Selleck Chemicals GmbH, Munich, Germany, at a stock concentration of 10 mM dissolved in dimethyl sulfoxide (DMSO). For experiments, Olaparib was diluted to 1.0 µM in cell culture medium to a final DMSO concentration of 0.1% (*v/v*). At 0.1% (14.3 mM), DMSO-related effects on treatment results can be excluded.

### 4.3. Ortho-IodoHoechst33258 (oIH)

The iodinated bis-benzimidazole, ortho-iodoHoechst 33258 (MedChemExpress, Beutelsbach, Germany), also termed as 2-(2-iodophenyl)-6-[6-(4-methylpiperazin-1-yl)-1H-benzimidazol-2-yl]-1H-benzimidazole (IUPAC), binds to DNA bases in the small groove of DNA. When exposed to UVA light, the molecule acts as a photosensitizer by generating highly reactive ligand radicals that cause DNA damage. The chemical was diluted in culture medium and adjusted to a concentration of 0.0075 µM for all experiments.

### 4.4. Radionuclides and External Irradiations

The radionuclide ^177^Lu[LuCl_3_], non-carrier-added, specific activity of 4110 GBq/mg was provided by the company ITG GmbH (Isotopen Technologies, Garching, Germany). The β-emitter ^177^Lu was characterized by a half-life of 6.64 days, a maximum β-energy of 497 keV and the most frequent photon energies of 208.4 keV and 113 keV. Radioactive labeling of (DOTA)[Tyr3, Thr8]-octreotide (DOTATATE; ABX, Radeberg, Germany) was performed in a reaction buffer (sodium acetate, gentisic acid) with 10 µg DOTATATE and 500 MBq ^177^Lu. The radiochemical product purity was determined by High-Performance Liquid Chromatography (HPLC) on a Shimadzu HPLC-system (Thermo Scientific, Dreieich, Germany) equipped with a reverse phase column (Merck Chromolith RP-18e). The radiochemical yield of the synthesis of ^177^Lu-DOTATATE (specific activity 80.6 MBq/nmol) was 99%, and the radiochemical purity was >95% for all syntheses.

^177^Lu[LuCl_3_] could not be used for the experiments because the carrier-free radionuclide showed strong adsorption on the laboratory vessels. This effect was avoided by using the radiopharmaceutical ^177^Lu[Lu]-DOTATATE, which ensured a nearly complete absence of both unlabeled ^177^Lu and unlabeled DOTATATE. Preliminary experiments with ^177^Lu[Lu]-DOTATATE have ruled out intracellular uptake in PC-3 cells. Thus, cellular binding or internalization could be excluded when ^177^Lu[Lu]-DOTATATE was applied. The radiation was emitted from the radioactive cell culture medium above the cell monolayer. Therefore, only the extracellular β-radiation was considered, and the term ”^177^Lu” is used in the following.

The α-particle emitter ^223^Ra-radium dichloride (^223^RaCl_2_, Xofigo) was provided by Bayer Vital GmbH (Leverkusen, Germany) with an activity concentration of 1000 kBq/mL. ^223^Ra (half-life 11.4 days) decayed through a cascade of short-lived α- and β-particle emitters. During each decay of ^223^Ra, four α-particles were produced, resulting in the emission of approximately 28 MeV of energy, with 95% of the energy coming from the α-emissions. Additionally, ^223^Ra emitted photons, e.g., γ-rays at 154, 269, 324 and 338 keV (intensities of 5.7%, 13.0%, 4.0% and 2.8%, respectively) [44].

An X-ray tube (Y.TU 320, Yxlon International, Dresden, Germany) with 200 kV X-rays (20 mA, dose rate ≈ 1.24 Gy/min, filtered with 0.5 mm Cu) was used for external irradiation.

The UV irradiation chamber BS-02 (Opsytec, Ettlingen, Germany) was used to expose the cells to UVA light (wavelength of 315–400 nm).

To compare the applied X-ray energy doses to the radioactivity, the activity of ^177^Lu and ^223^Ra corresponding to the equivalent X-ray dose was calculated. The calculation was performed with Geant4 simulations for a 10 µm cell monolayer at the bottom of the well (9.6 cm^2^) in 2 mL cell culture medium. According to this model, only the extracellular irradiation of the medium was considered [45].

### 4.5. Irradiation Procedures

To evaluate the cytotoxicity of Olaparib , the PC-3 cells were plated in 6-well multititer plates (MTP) on day zero. The next day, Olaparib was added in the concentration range of 0.01–50 µM. On day one or five, the cells were trypsinized and plated for the clonogenic survival assays. Considering the cytotoxicity of Olaparib for the combined treatment of PC-3 cells, 1 µM Olaparib was used, which confirms to the inhibitor concentration IC_30_.

For the combination experiments, the PC-3 cells were plated in 6-well MTP one day prior to the irradiation experiments. Exceptionally, the oIH+UVA experiments were performed in 24-well MTP. To compare the dose–response relationship of co-treatment with Olaparib and ^177^Lu or ^223^Ra, PC-3 cells were incubated in 6-well MTP with radioactive solutions of 0.1–10 MBq/mL culture medium and 0.5–10.8 kBq/mL for 24 h, respectively, to achieve doses of 0.1–8.5 Gy or 0.1–2.0 Gy. For the X-ray irradiation, the cells were exposed to 1.0–10.0 Gy corresponding to maximum exposure times of approximately eight minutes at 10.0 Gy.

To investigate the cytotoxic effects of the photodynamic approach (oIH + UVA), the cells were pretreated with 7.5 × 10^−3^ μM oIH for one hour. The cells were then irradiated in 24-well MTP in the absence or presence of Olaparib (1 μM) with UVA doses of 1.0–7.0 J/cm^2^ at room temperature. Untreated control samples were included in each experiment.

After each exposure, the culture medium was replaced with fresh medium (37 °C). Cells were then detached by trypsinization and further treated without or with Olaparib for 5 or 12 days in 6-well MTP.

### 4.6. γH2A.X Foci Analysis

Immunostaining was performed as previously described by Maucksch et al. [46]. Briefly, aliquots of irradiated cells were centrifuged by cytospin (Universal 30 RF, Hettich, Tuttlingen, Germany) onto glass slides, fixed in 4% (*v/v*) formaldehyde (Merck Chemicals, Darmstadt, Germany), and permeabilized using Triton X100 (Merck). After blocking the cells with 1% (*v/v*) bovine serum albumin (BSA; Sigma Aldrich, Taufkirchen, Germany) in PBS, mouse monoclonal anti-phospho-histone H2A.X antibody (Merck) was added. The cells were then incubated with goat anti-mouse IgG conjugated to Alexa Fluor 488 (ThermoFisher, Darmstadt, Germany) for 1 h. To stain DNA, 4.6’-diamidino-2-phenylindole (DAPI, Sigma Aldrich) was added, and after washing, the cell spots were embedded in mounting medium (Dako Cytomation, Glostrup, Denmark). Three replicates of slides were made for each dose point.

The γH2A.X foci were automatically evaluated using the AKLIDES^®^ platform (Medipan, Dahlewitz, Germany), which consists of modules for image analysis with pattern recognition algorithms. DAPI was used as a fluorescent dye for focusing, quality assessment and object recognition. Subsequently, the immunofluorescence of the detected cells was captured in three z-layers [38].

### 4.7. Cell Cycle Analysis

Cell cycle analysis was performed by fluorescence activated cell sorting (FACS) to quantify the cell cycle phases (Accuri C6plus, Becton Dickinson GmbH, Heidelberg, Germany).

Cells were irradiated as described for colony formation analyses. After 5 days of incubation with Olaparib and radionuclides, the culture medium was removed, and the cells were further processed for cell cycle analysis according to a standard protocol [47]. Briefly, the cells were trypsinized, transferred to centrifugation tubes, washed with PBS and placed on ice. For fixation, methanol 70% (*v/v*) was added, and the cells were stored at −20 °C. After fixation, the cells were washed with PBS and incubated with a solution of RNAse (Sigma Aldrich), 5% in PBS and stained with propidium iodide (PI) 40 µg/mL in PBS (Sigma Aldrich) for 30 min at 4 °C.

Samples were measured using an Accuri^®^ C6 plus flow cytometer (Becton Dickinson Biosciences, Franklin Lakes, NJ, USA) and analyzed using FlowLogic™ 8.7 software.

### 4.8. Statistics

The results of the colony formation assay are represented as mean values and SD (standard deviation) based on three independent experiments, with each experimental condition considered a triplicate.

The γH2AX-foci assay, cell cycle and sub-G1 analysis have been conducted two or three times. The mean values and standard deviations (SD) or the standard error of the mean (SEM) for the cell cycle and sub-G1 are displayed.

Statistical analysis of the experimental results was performed using a two-tailed, unpaired student *t*-test with GraphPad Prism version 10.2.3.403. A difference between two independent samples was considered significant at a probability of error of *p* ≤ 0.05.

## 5. Conclusions

In this study, we showed an increased eradication of PC-3 cells when combining Olaparib with different types of radiation. Although cytotoxic effects varied depending on the type of radiation, the strongest effects were observed for the low-LET radiation of ^177^Lu, applicable for all biological assays. Similar radiosensitization effects in clonogenic survival were found for ^223^Ra, X-rays and the PDT. However, the γH2A.X foci assay and the sub-G1 analysis identified cytotoxic effects of ^177^Lu, ^223^Ra and PDT combined by Olaparib in more detail.

In light of the growing impact of Olaparib in clinical settings and its recent approval for use in prostate cancer, it is crucial to explore the optimal combinations that can yield therapeutic benefits. This study underlines the need for scientific comparisons at the cellular level to optimize the use of radiopharmaceuticals. The results presented herewith may have significant therapeutic implications, as they offer preliminary insights into potential clinical applications of combination therapies in nuclear medicine. Given the increasing use of targeted radionuclide therapies, such as PRLT in prostate cancer, where ^177^Lu-PSMA and ^225^Ac-PSMA represent the most commonly employed radiopharmaceuticals, it is imperative to evaluate whether the usage of PARPi can improve the efficacy of this therapeutic modality.

## Figures and Tables

**Table 1:** Overview of the calculated D_37_-values, including their confidence intervals and dose-modifying factors (DMF) at the respective D_37_-values.

**Calculated Parameters**	**^177^** **Lu**	**^177^** **Lu + Olaparib 5d**	**^177^** **Lu + Olaparib 12d**
D_37_ (Gy)	2.25	1.28	1.82
Confidence Interval (Gy)	2.020–2.486	1.093–1.477	1.412–2.253
DMF	1	1.77	1.24
	**^223^** **Ra**	**^223^** **Ra + Olaparib 5d**	**^223^** **Ra + Olaparib 12d**
D_37_ (Gy)	0.32	0.27	0.28
Confidence Interval (Gy)	0.300–0.331	0.260–0.280	0.258–0.292
DMF	1	1.17	1.15
	**X-ray**	**X-ray + Olaparib 5d**	**X-ray + Olaparib 12d**
D_37_ (Gy)	4.34	3.74	3.36
Confidence Interval (Gy)	3.902–4.687	3.270–4.124	3.121–3.583
DMF	1	1.16	1.11
	**oIH + UVA**	**oIH** **+ UVA + Olaparib 5d**	**oIH** **+ UVA + Olaparib 12d**
D_37_ (J/cm^2^)	2.23	1.79	1.43
Confidence Interval (J/cm^2^)	1.886–2.522	1.432–2.122	1.123–1.771
DMF	1	1.25	1.25

**Table 2:** Radiosensitizing effects of Olaparib combined with various radiation sources on PC-3 cells. Factors were calculated by the number of γH2A.X foci for irradiation in the presence of Olaparib in relation to the number of γH2A.X foci in the absence of Olaparib at the indicated dose points.

Biological Assay	Radiosensitizing Effects of Olaparib						
	^177^Lu		^223^Ra		oIH + UVA		X-ray
	Dose (Gy)		Dose (Gy)		UVA-Dose (J/cm^2^)		
	3.2	6.4	0.75	1.5	3.0	7.0	
γH2A.X foci assay ^2^	3.27 ± 0.82	4.15 ± 1.78	2.14 ± 0.95	0.79 ± 0.25	2.86 ± 2.70	1.59 ± 0.43	n.a ^1^

^1^ Not applicable. ^2^ Enhancement factors ± SEM.

**Table 3:** Radiosensitizing effects of Olaparib combined with various radiation sources on PC-3 cells. Factors were calculated by the relation of cell cycle distributions (%) and sub-G1 fractions (%) for irradiation in the presence and absence of Olaparib at the indicated dose points.

Biological Assay	Radiosensitizing Effects of Olaparib			
	^177^Lu		^223^Ra	
	Dose (Gy)		Dose (Gy)	
	3.2	6.4	0.75	1.5
Cell cycle Distribution G2/M ^1^	1.25 ± 0.43	1.86 ± 1.15	1.30 ± 0.33	0.88 ± 0.08
Sub-G1 fraction ^1^	3.13 ± 0.25	1.76 ± 0.12	1.39 ± 0.10	1.19 ± 0.11

^1^ Enhancement factors ± SEM are displayed.

## Data Availability

The data can be requested by contacting the corresponding author.
